# Protein crystal structure from non-oriented, single-axis sparse X-ray data

**DOI:** 10.1107/S2052252515018795

**Published:** 2016-01-01

**Authors:** Jennifer L. Wierman, Ti-Yen Lan, Mark W. Tate, Hugh T. Philipp, Veit Elser, Sol M. Gruner

**Affiliations:** a Field of Biophysics, Cornell University, Ithaca, NY 14853, USA; b Cornell High Energy Synchrotron Source (CHESS), Cornell University, Ithaca, NY 14853, USA; c Laboratory of Atomic and Solid State Physics, Cornell University, Ithaca, NY 14853, USA

**Keywords:** X-ray serial microcrystallography, sparse data, *EMC* algorithm, protein microcrystallography, synchrotron-radiation sources

## Abstract

X-ray free-electron lasers (XFELs) have inspired the development of serial femtosecond crystallography (SFX) as a method to solve the structure of proteins. SFX datasets are collected from a sequence of protein microcrystals injected across ultrashort X-ray pulses. The idea behind SFX is that diffraction from the intense, ultrashort X-ray pulses leaves the crystal before the crystal is obliterated by the effects of the X-ray pulse. The success of SFX at XFELs has catalyzed interest in analogous experiments at synchrotron-radiation (SR) sources, where data are collected from many small crystals and the ultrashort pulses are replaced by exposure times that are kept short enough to avoid significant crystal damage. The diffraction signal from each short exposure is so ‘sparse’ in recorded photons that the process of recording the crystal intensity is itself a reconstruction problem. Using the *EMC* algorithm, a successful reconstruction is demonstrated here in a sparsity regime where there are no Bragg peaks that conventionally would serve to determine the orientation of the crystal in each exposure. In this proof-of-principle experiment, a hen egg-white lysozyme (HEWL) crystal rotating about a single axis was illuminated by an X-ray beam from an X-ray generator to simulate the diffraction patterns of microcrystals from synchrotron radiation. Millions of these sparse frames, typically containing only ∼200 photons per frame, were recorded using a fast-framing detector. It is shown that reconstruction of three-dimensional diffraction intensity is possible using the *EMC* algorithm, even with these extremely sparse frames and without knowledge of the rotation angle. Further, the reconstructed intensity can be phased and refined to solve the protein structure using traditional crystallographic software. This suggests that synchrotron-based serial crystallography of micrometre-sized crystals can be practical with the aid of the *EMC* algorithm even in cases where the data are sparse.

## Abstract 2

Using the *EMC* algorithm, the three-dimensional intensity was successfully reconstructed from millions of non-oriented, sparse data frames collected from a hen egg-white lysozyme crystal rotating about a single axis. The protein structure was solved from the reconstructed intensity. This result is encouraging for the development of synchrotron-based serial microcrystallography.

## 1. Introduction   

The advent of X-ray free-electron lasers (XFELs) has catalyzed interest in obtaining the atomic structures of proteins from sequentially exposed microcrystals. The scientific motivation is that protein crystallization is still the major bottleneck in structural studies, and it may well be that many, if not most, important protein systems may be more readily crystallized in the form of numerous microcrystals of micrometre or submicrometre sizes (Gati *et al.*, 2014 ▸; Hunter & Fromme, 2011 ▸; Nederlof *et al.*, 2013 ▸; Quevillon-Cheruel *et al.*, 2004 ▸; Weierstall *et al.*, 2014 ▸). The approach that has been taken with XFELs is serial femtosecond crystallography (SFX) based on the ‘diffract-before-destroy’ principle (Neutze *et al.*, 2000 ▸). In SFX experiments, datasets are collected from randomly oriented microcrystals injected sequentially across ultrashort pulses of an XFEL and recorded using a fast-framing detector (Philipp *et al.*, 2008 ▸). Each X-ray pulse is sufficiently short in duration (tens of femtoseconds) that it is diffracted and exits the crystal before the crystal is vaporized into plasma by electron ejection. The high peak intensities of XFELs allow strong sufficient diffraction from each crystal so that the crystal orientation can be determined by indexing individual frames. Reflections can then be integrated using, for example, Monte Carlo integration in the *CrystFEL* suite (White *et al.*, 2012 ▸).

Although XFELs are becoming more prevalent, XFEL beam time is expected to continue to be very limited for at least a decade. The success of SFX has catalyzed experiments with the goal of performing serial crystallography with small crystals at the more prevalent and readily accessible storage-ring synchrotron-radiation (SR) sources (Gati *et al.*, 2014 ▸; Stellato *et al.*, 2014 ▸; Botha *et al.*, 2015 ▸; Nogly *et al.*, 2015 ▸). On optimized SR source beamlines the exposure time of each crystal will be in the millisecond to submillisecond range, thereby enabling structural experiments of practical (minutes to hours) duration, even for crystals that are not cryocooled (for a detailed discussion of serial crystallography at SR sources, see Gruner & Lattman, 2015 ▸). The goal is to acquire complete datasets by merging diffraction data from a succession of tiny crystals, the total volume of which is practically comparable to that of a single large crystal.

At SR sources, the number of diffracted photons in a given exposure from a microcrystal is limited by the dose that can be tolerated before classic radiation damage compromises the diffraction (Nave & Garman, 2005 ▸; Nave & Hill, 2005 ▸). Smaller crystals yield fewer diffracted photons. Ultimately, a sufficiently small crystal size is reached such that the number of photons diffracted per frame is too small to resolve Bragg peaks. We call such X-ray exposures ‘sparse’. Intuitively, one might believe that sparse exposures can never be merged into complete datasets. However, this has already been shown not to be the case for a nonprotein structure (Ayyer *et al.*, 2015 ▸). Below, we demonstrate that this is also the case for a protein crystal.

The *EMC* algorithm (Loh & Elser, 2009 ▸), which was originally developed for single-particle imaging experiments at XFELs, suggests that complete datasets can still be determined from the unindexable frames, if enough measurements or frames are available. An expectation-maximization scheme is applied by the *EMC* algorithm to update the probability distribution of orientations of each frame iteratively, and the redundancy in the large number of measurements is sufficient for a unique reconstruction. Orientation recovery from sparse, non-oriented frames using the *EMC* algorithm has been demonstrated in two-dimensional shadowgraphy, three-dimensional shadowgraphy and crystallography with an inorganic crystal (Philipp *et al.*, 2012 ▸; Ayyer *et al.*, 2014 ▸, 2015 ▸).

**Figure 1:**
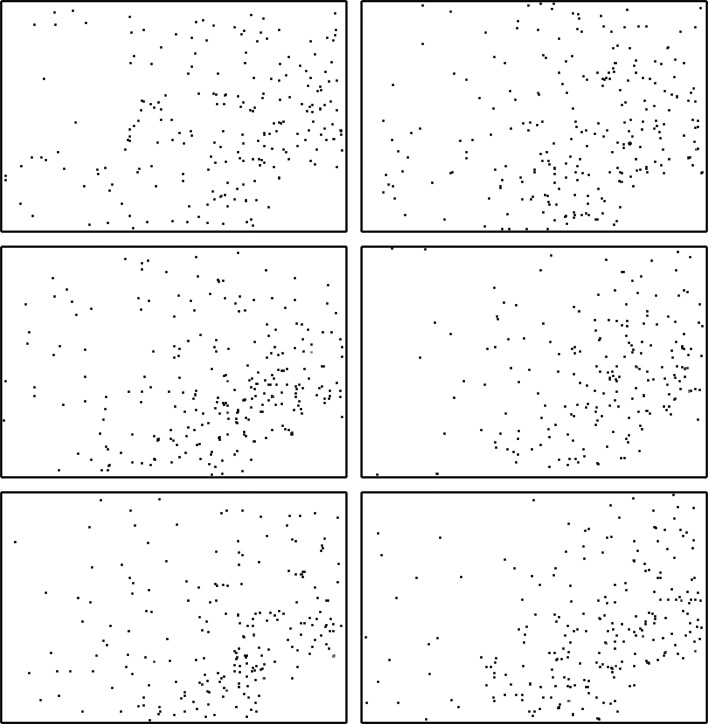
Random selection of six data frames (393 × 262 pixels). The direct beam is incident normally at the lower right region of the detector, which is blocked by the beamstop. The resolution at the upper left corner is 1.3 Å. Each frame consists of only ∼200 photons on average and the maximal photon count in these frames is three per pixel. The size of the pixels is smaller than the rendered photons in this image, which are enlarged for visual clarity.

In this proof-of-principle study, we collected eight million sparse frames from a rotating hen egg-white lysozyme (HEWL) crystal of 400 µm in size with a relatively dim laboratory X-ray source and the fast-framing Mixed-Mode Pixel Array Detector (MM-PAD; Tate *et al.*, 2013 ▸) to simulate frames collected from microcrystals at storage-ring sources. Each frame consists of ∼200 photons on average (Fig. 1 ▸). With only the prior knowledge of the unit-cell parameters and the rotation-axis orientation, we successfully reconstructed the three-dimensional Bragg intensities of the crystal. The algorithm made no assumptions about the crystal symmetry and was not given the angle of each frame about the rotation axis. Our reconstructed intensities were of sufficient quality for a molecular-replacement phasing algorithm to solve the structure to 1.5 Å resolution.

## 2. Methods   

### 2.1. Sample preparation   

Lyophilized lysozyme powder from hen egg white (Sigma, St Louis, Missouri, USA) was used for crystallization by dissolving it in deionized water to 50 mg ml^−1^ without further purification. Crystals were grown at 293 K in 6 µl droplets by the hanging-drop diffusion method with a 50% buffer solution consisting of 1.0 *M* sodium chloride plus 0.1 *M* sodium acetate at pH 4.5 with 20% PEG. Crystals were retrieved from the droplets after maximum growth after a few days with a Hampton Research CryoLoop. Crystals were then mounted on a goniometer, flash-cooled under an Oxford Cryosystem Cryostream and kept at 100 K for data collection. By cryocooling a single macrocrystal, we mimicked an experiment with multiple microcrystals that are discarded as they become damaged.

### 2.2. Data collection   

**Figure 2:**
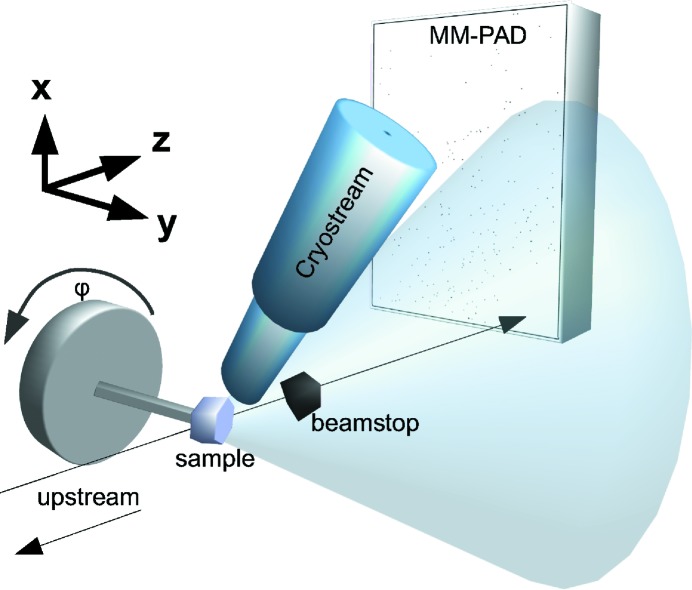
A simplified schematic of the experimental setup with the X-ray beam originating from the left side of the image along the *z* axis. It illuminates the crystal rotating about the *y* axis (or 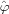), perpendicular to the beam axis. The main beam is then blocked by a beamstop. The diffracted photons are recorded with the MM-PAD. A cryostream (in blue) cools and maintains the crystal at 100 K. The figure is not drawn to scale.

A single HEWL crystal of approximately 400 µm in size was mounted on the goniometer and set continuously rotating on a rotation stage (Newport URS100) at 0.05° per second. The axis of rotation was set to be perpendicular to the beam axis during data collection, as shown in Fig. 2 ▸. The sample was illuminated by a Cu *K*α X-ray beam (1.54 Å wavelength) generated from a rotating anode set to 40 kV and 50 mA (Rigaku RU-H3R). The X-ray beam, with a flux of 10^7^ photons s^−1^, was focused to a ∼0.5 × 0.5 mm^2^ spot at the sample using Ni-coated Franks mirrors placed 1 m from the sample. The beam had a divergence of 1 mrad. Sparse data frames were ensured by simply reducing the exposure time per frame to a sufficiently short duration. An MM-PAD at a distance of 33 mm from the rotating sample recorded frames with a 10 ms exposure time, providing a 0.0005° oscillation angle per frame. The center of the beam was placed in one corner of the active area of the MM-PAD to record the highest possible resolution, which was approximately 1.3 Å. A pin-diode beamstop was used to keep the direct beam from striking the detector while recording the intensity.

The data frames were then thresholded and photon counts were obtained using a procedure similar to that employed by Ayyer *et al.* (2014 ▸, 2015 ▸). A data set of 8.8 million frames, which corresponds to 12 full revolutions of the crystal, with an average of ∼200 photons per frame was then passed to the *EMC* algorithm. Although we knew the orientation of each data frame, this information was not used by the *EMC* procedure.

### 2.3. Orientation recovery   

#### 2.3.1. *EMC* algorithm   

We used the *EMC* algorithm developed by Loh & Elser (2009 ▸) to iteratively assemble the non-oriented, shot-noise-limited frames into a three-dimensional intensity map. Each iteration consists of three steps: expansion (E), maximization (M) and compression (C). Starting with an initial intensity estimate *W*(**q**), with spatial frequency denoted by **q**, the expansion step samples slices of *W*(**q**) for crystal orientations Ω_*j*_. Intensity slices are arrays *W_ij_* of average photon counts at pixel *i* when the crystal has orientation Ω_*j*_. Further, we define *P_jk_*(*W*) as the conditional probability, based on the intensity *W*(**q**), that the crystal had orientation Ω_*j*_ in data frame *k*. The data in frame *k* are the photon counts *K_ik_* at each pixel *i*. Assuming a uniform distribution over the set of possible orientations, independent Poisson statistics on the photon counts at each pixel gives us the following formula for the conditional probability:
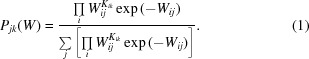
In the maximization stage, the average photon counts *W_ij_* are updated by maximizing the likelihood function associated with *P_jk_*(*W*) with the rule
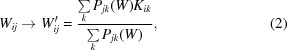
which has the simple interpretation as the expected photon count according to the probability distribution *P_jk_*(*W*). The compression step subsequently maps the updated *W* slices back to a new three-dimensional intensity *W*′(**q**), which ensures the consistency of intensity slices in the next round. Using this scheme, the *EMC* algorithm searches for the most probable intensity distribution that is consistent with all of the data frames.

#### 2.3.2. Rotation-group sampling   

Because the experimental setup only allows orientation sampling within a small rotation subspace, one can expect difficulty in searching for a solution within the whole rotation space, unless the constraint imposed by the measurement is strong, which is not the case in the sparse regime. Therefore, we confined ourselves to a uniform distribution of one-dimensional rotations about the rotation axis in this study. We note that crystals generally will have random orientations over all three-dimensional rotations in serial crystallography. This broader rotation-angle space will be explored in future studies. Since frames were taken sequentially while rotating, we merged the first revolution into bins of width 1° to retrieve the rotation-axis orientation with the *XDS* package (Kabsch, 2010 ▸).

#### 2.3.3. Seeding   

To test the robustness of the *EMC* algorithm, we assumed that the parameters of the tetragonal unit cell were only known roughly, as might be the case, for example, from a diffraction powder pattern. The initial intensity estimate was seeded by placing small three-dimensional Gaussian peaks of random height at each predicted Bragg position. In principle *EMC* should be able to reconstruct the intensity profiles starting from a random model, as described in Loh & Elser (2009 ▸), but the highly discontinuous diffraction from crystals disrupts the convergence of the reconstruction. The reconstruction converged when seeding delta functions of random height at predicted positions. However, we found that seeding with random Gaussian peaks worked much better because it incorporates the finite sizes of the reflections. No symmetry, such as Friedel pairs or systematic absences, was imposed in this process.

### 2.4. Integration   

The *EMC* algorithm reconstructs the total scattered intensity, including the diffuse background scatter, which should be subtracted from the Bragg peak intensities. In addition, the Bragg peaks do not necessarily fall perfectly onto any *a priori* lattice. To determine precise values of the reciprocal-lattice constants, we use a three-dimensional version of the peak-segmentation algorithm described in Zhang *et al.* (2006 ▸). The algorithm proceeds for several iterations, and each iteration refines the segmentation from the previous iteration. The segmentation is a classification of voxels into signal or background based on a standard score. The standard score *z*(*W*) of a voxel with intensity value *W* is computed as
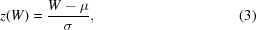
where μ and σ are the mean and standard deviation, respectively, of the voxels in a surrounding *n* × *n* × *n* cube. Voxels with standard score above a particular threshold γ are classified as signal. This procedure is repeated three more times with the difference that the μ and σ computation only includes the voxels classified as background in the previous iteration. For good-quality segmentation of the Bragg peaks, we increased γ from 1.0 to 3.0 in successive iterations. For a candidate set of reciprocal-lattice constants, we computed the total intensity of segmented peaks lying within ellipsoids centered on the corresponding Bragg positions. The ellipsoid volume was a small fraction of the reciprocal unit cell, with principal axes consistent with the tetragonal cell. The reciprocal-lattice constants giving the greatest total intensity were taken as the refined values.

Using the refined reciprocal-lattice constants, we determined the Bragg peak intensities using the following integration procedure. An ellipsoid window is centered on each Bragg peak. If a voxel is within such a window, it is assigned to the corresponding peak; otherwise, it is classified as background. These ellipsoids were similar to those used in parameter refinement but were larger, increasing from 10 to 50% of the reciprocal-cell volume. The mean of the background voxels were then subtracted from each signal voxel before being summed to give the intensity for each reflection. Partial peaks, such as those adjacent to boundary, detector gaps or the beamstop region, were rejected.

### 2.5. Phasing, model building and refinement   

The reconstructed intensities and subsequent structure factors were fed into *MOLREP* (Vagin & Teplyakov, 2010 ▸) from the *CCP*4 suite (Winn *et al.*, 2011 ▸) to produce molecular-replacement solutions using several published coordinates of lysozyme from the Protein Data Bank (PDB entries 193l, 1flq, 1lz1 and 2lzm; Vaney *et al.*, 1996 ▸; Masumoto *et al.*, 2010 ▸; Artymiuk & Blake, 1981 ▸; Weaver & Matthews, 1987 ▸) as starting models. We refined the solutions through 20 iterations in *REFMAC* (Murshudov *et al.*, 2011 ▸) with both rigid-body and restrained refinement and rebuilt them in *Coot* (Emsley *et al.*, 2010 ▸) with cyclical refinement. Refinement statistics are shown for 193l in Table 1 ▸, as the final molecular-replacement solution used 193l as the model for phasing. Structure 193l has the sequence of the HEWL crystal used for reconstruction and provided the highest contrast for a solution in *MOLREP*. To test the limits of our reconstructed data within molecular-replacement phasing solutions, we used several different forms of lysozyme for the phasing model with varying results. PDB entry 1flq is a mutant of HEWL with all alanines substituted by glycine and has 99.2% similarity to 193l. While *MOLREP* provided solutions for our reconstructed data with phases from 1flq, the refined map is less ordered and fits more poorly with 193l. Next, we used PDB entry 1lz1, a human lysozyme with one additional residue and only 76.9% similarity to 193l. This structure also provided molecular-replacement solutions with less contrast and that fitted more poorly with 193l. Lastly, we used PDB entry 2lzm, a bacteriophage lysozyme with a similarity of only 21.0% and 35 more residues than HEWL, for molecular replacement. Here, *MOLREP* did not provide a phased solution for our reconstruction.

## 3. Results   

### 3.1. Validation of reconstruction   

**Figure 3:**
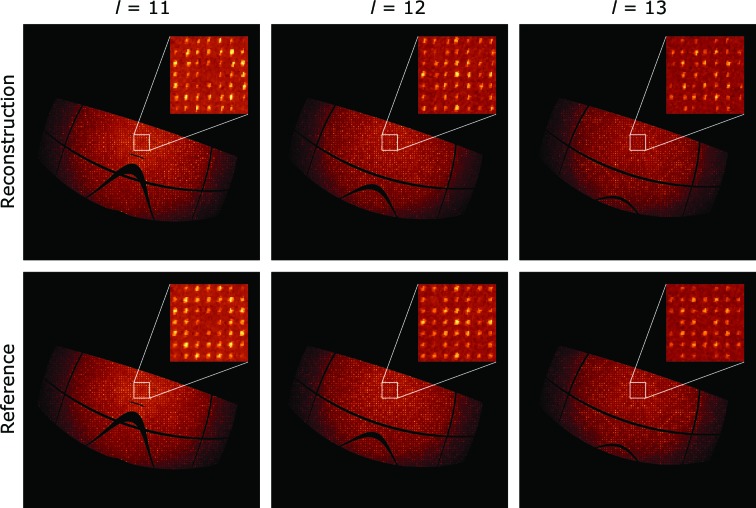
Slices of the reconstructed and reference intensity maps in the *hk* plane at constant values of *l*. Even without imposing symmetry when seeding the initial intensity estimate, the reconstructed intensity obeys the reflection condition 00*l*: *l* = 4*n* required by the *P*4_3_2_1_2 space-group symmetry of the HEWL crystal (see insets). The mapping into reciprocal space transforms the detector gaps (Tate *et al.*, 2013 ▸) into curves.

As a check, the reconstructed intensity distribution in reciprocal space was compared with the actual intensity distribution. The actual (*i.e.* ‘reference’) distribution could be recovered because the orientation of each frame was known, even though this information was not used in the *EMC* reconstruction. Several slices of the reconstructed intensity and reference intensity perpendicular to the *l* axis are shown in Fig. 3 ▸. We checked that the reconstructed intensity obeys the reflection conditions 00*l*: *l* = 4*n* and *h*00: *h* = 2*n* required by the *P*4_3_2_1_2 space-group symmetry of the HEWL crystal (Hahn, 2006 ▸). This suggests a successful orientation recovery because no symmetry was imposed when we seeded the initial intensity estimate.

**Figure 4:**
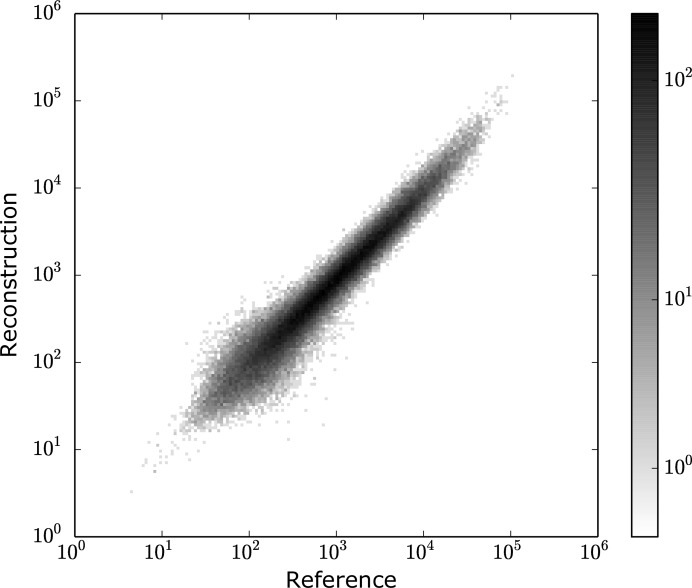
Scatter plot comparing the reconstructed Bragg peak intensities with the reference intensities.

A more direct justification involves comparing the integrated reflections. Using
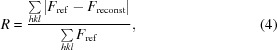
where *F*_ref_ and *F*_reconst_ are the structure factors of the reference intensity and reconstructed intensity, respectively, we calculated that our reconstructed intensity has *R* = 4.73% compared with the reference intensity. Fig. 4 ▸ shows a scatter plot comparing the reconstructed intensities with the reference intensities. The reflections collapse well on the diagonal, which indicates that the orientations of most frames were recovered by the *EMC* algorithm. We expect that the distribution of reflections in the scatter plot becomes broader as the average photon count per frame decreases, because this reduces the information for orientation recovery.

**Figure 5:**
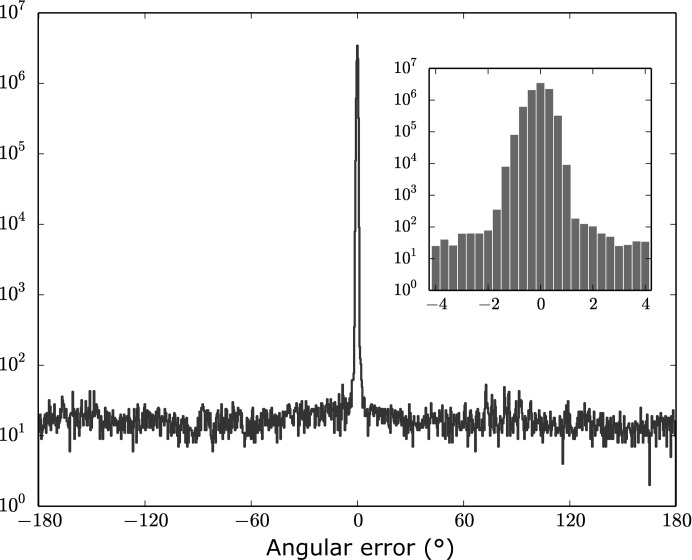
Histogram of the difference between the most probable orientations of frames and the actual orientations, expressed in degrees about the rotation axis. The *EMC* algorithm correctly assigned 99.7% of the frames within 1°, as shown in the inset.

The difference between the most probable orientation of each frame assigned by the *EMC* algorithm and its actual orientation is shown in Fig. 5 ▸ as a histogram of one-dimensional rotations about the rotation axis. We found that 99.7% of the frames were assigned to the correct orientation within 1°. We suspect that the outliers are owing to an abnormally low signal-to-noise ratio in some frames, perhaps caused by extra background scatter from the cryoloop or an orientation with few reflections. This motivates the necessity of background reduction in future experiments, specifically in the case of small or weakly diffracting crystals.

### 3.2. Validation of structure   

**Figure 6:**
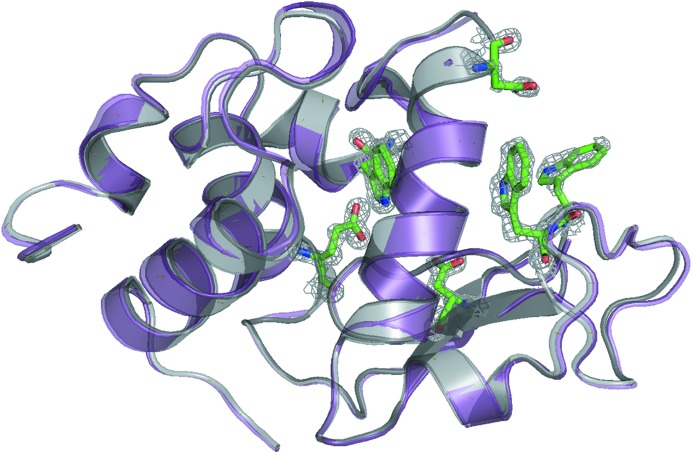
Structure of the reconstructed protein (gray) compared with the model 193l (purple) used in molecular replacement. Comparison of higher resolution features (active sites) are rendered as green sticks (model structure) and gray mesh (reconstruction).

The structure we built from the *EMC*-reconstructed intensities (Fig. 6 ▸) agrees with the published structure of lysozyme with PDB entry 193l. The r.m.s. difference when all of the C^α^ atoms of the two structures are superimposed is 0.27 Å, which could be attributed to differing solvent content during crystallization and water placement during refinement between the deposited model and our initial crystal. With a completeness of 92.01%, 16 056 independent reflections, an *R* factor of 0.28 and an *R*_free_ of 0.32, our structure determined from reconstructed sparse data compares favorably with structures obtained by more conventional means.

### 3.3. Validation of sparse data   

**Figure 7:**
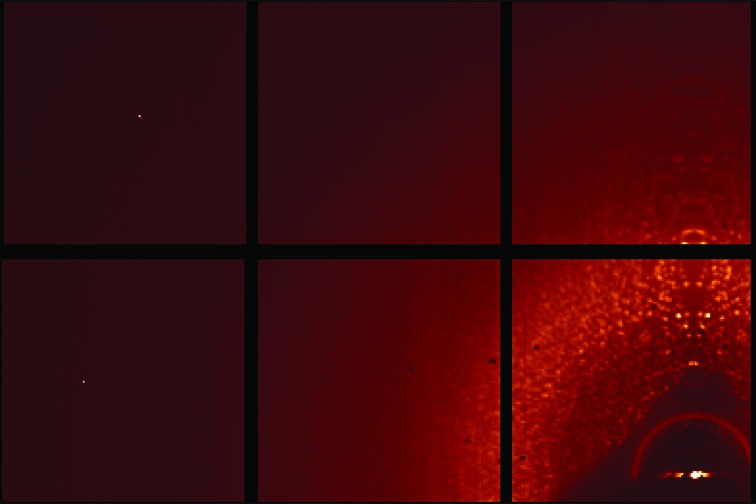
From the sum of all of the frames, one can see diffuse scatter owing to solvent, air and windows, while discernible peaks die out at wider scattering angles. The rotation axis is almost parallel to the vertical direction in this image and therefore the sum seems symmetric about the vertical direction.

From the reconstructed intensity map, we were able to identify regions that were not beneath Bragg peaks and integrate these to determine that about 80% of the counts were background photons that did not fall beneath Bragg peaks. This can be seen from the sum of all of the frames (Fig. 7 ▸), where reflections at wider scattering angles are indistinguishable from the diffuse background. The fact that the majority of the photons reaching the detector in these sparse data frames originate from background sources is why conventional methods fail to identify Bragg peaks. This lack of sensitivity to background is special to crystal datasets and is consistent with the findings of Ayyer *et al.* (2015 ▸).

### 3.4. Computational details   

We performed the reconstruction on a single machine (Intel Xeon E5-2640, 2.00 GHz, with 128 GB RAM running Scientific Linux) using 16 cores. The estimates of unit-cell parameters were *a* = *b* = 77.0, *c* = 36.0 Å, and the reconstruction used data up to a resolution of 2.0 Å, with only 195 photons per frame on average. We used a reciprocal-lattice grid with voxel size a*/7, which corresponds to 543 × 543 × 543 voxels. The sampled rotations consisted of 1080 uniformly distributed rotations about the rotation axis. The reconstruction ran for 30 iterations and each iteration took 1.3 h on average. Convergence was monitored by the r.m.s. change of the three-dimensional intensities, which was found to be insensitive to the choice of random seeds for the initial intensities. Based on the converged intensity at 2.0 Å resolution, the probability distribution *P_jk_*(*W*) was calculated and fed into (2) to incorporate data up to 1.3 Å resolution. For this we used a finer reciprocal grid of size a*/9 (939 × 939 × 939 voxels) to mitigate peak overlaps. The resulting intensity was rescaled so that its sum equals the total number of recorded photons over all of the frames; this is what we call the reconstructed intensity. We used *n* = 15 for the size of the cubic window in peak segmentation, as described in §[Sec sec2.4]2.4. The Bragg peak intensities were integrated using the refined unit-cell parameters *a* = *b* = 77.52, *c* = 36.23 Å.

## 4. Conclusion   

Here, we have shown experimentally that a series of non-oriented, sparse diffraction frames from a protein crystal rotating about a single rotation axis can be assembled into a three-dimensional intensity with the aid of the *EMC* algorithm. Validation of reconstruction is supported by the recovery of symmetries which were absent in the initial seeding process, the consistency of integrated reflections with the reference intensity and the comparison of the most probable orientations of frames with the actual orientations. Moreover, we have demonstrated that the protein structure can be solved by phasing the integrated reflections of the reconstruction through molecular replacement. This result suggests that the indexability of each frame *per se* does not necessarily limit structure determination in serial crystallo­graphy.

In fact, this study may relax many limitations in serial crystallography imposed by indexability of frames: *i.e.* the size of the crystal, the brilliance of the X-ray source or radiation sensitivity. With minor modifications, one can envision a serial microcrystallography experiment performed at room temperature at storage-ring sources within microfluidic chips (Heymann *et al.*, 2014 ▸) or from gel injectors (Nogly *et al.*, 2015 ▸; Weierstall *et al.*, 2014 ▸). Several features are still needed to make our experiment more realistic for serial crystallography. One is the sampling of the entire rotation group, in which the constraint for solution convergence shall be stronger because of the larger redundancy among frames. The computation time, which scales with the product of the number of rotations and the number of frames, is expected to grow rapidly at the same time, so further optimizations are necessary. Also, background reduction, such as the usage of a graphene window (Wierman *et al.*, 2013 ▸), is desirable when obtaining data from multiple small crystals. For the future, we plan proof-of-principle experiments in which the entire rotation group is sampled and data are collected from multiple crystals. If successful, serial microcrystallography should be feasible at storage-ring sources even from crystals that are so small that single indexable exposures cannot be obtained.

## Figures and Tables

**Table 1:** Refinement statistics

Reconstruction	
Space group	*P*4_3_2_1_2
Unit-cell parameters (Å)	*a* = *b* = 77.5, *c* = 36.2
Resolution (Å)	54.801–1.497
Completeness (%)	92.01
No. of independent reflections	16056
Refinement	
No. of atoms	1963
*R* factor	0.2823
*R*_free_	0.3199
R.m.s.d., bond lengths (Å)	0.0192
R.m.s.d., bond angles (°)	0.1200
